# Molecular Mapping of *PMR1*, a Novel Locus Conferring Resistance to Powdery Mildew in Pepper (*Capsicum annuum*)

**DOI:** 10.3389/fpls.2017.02090

**Published:** 2017-12-08

**Authors:** Jinkwan Jo, Jelli Venkatesh, Koeun Han, Hea-Young Lee, Gyung Ja Choi, Hee Jae Lee, Doil Choi, Byoung-Cheorl Kang

**Affiliations:** ^1^Department of Plant Science, Plant Genomics and Breeding Institute, and Vegetable Breeding Research Center, College of Agriculture and Life Sciences, Seoul National University, Seoul, South Korea; ^2^Center for Eco-friendly New Materials, Korea Research Institute of Chemical Technology, Daejeon, South Korea; ^3^Department of Plant Science, College of Agriculture and Life Sciences, Seoul National University, Seoul, South Korea

**Keywords:** *Capsicum annuum*, *Leveillula taurica*, marker-assisted selection, molecular markers, *PMR1*, powdery mildew resistance

## Abstract

Powdery mildew, caused by *Leveillula taurica*, is a major fungal disease affecting greenhouse-grown pepper (*Capsicum annuum*). Powdery mildew resistance has a complex mode of inheritance. In the present study, we investigated a novel powdery mildew resistance locus, *PMR1*, using two mapping populations: 102 ‘VK515' F_2:3_ families (derived from a cross between resistant parental line ‘VK515R' and susceptible parental line ‘VK515S') and 80 ‘PM Singang' F_2_ plants (derived from the F_1_ ‘PM Singang' commercial hybrid). Genetic analysis of the F_2:3_ ‘VK515' and F_2_ ‘PM Singang' populations revealed a single dominant locus for inheritance of the powdery mildew resistance trait. Genetic mapping showed that the *PMR1* locus is located on syntenic regions of pepper chromosome 4 in a 4-Mb region between markers CZ2_11628 and HRM4.1.6 in ‘VK515R'. Six molecular markers including one SCAR marker and five SNP markers were localized to a region 0 cM from the *PMR1* locus. Two putative nucleotide-binding site leucine-rich repeat (NBS-LRR)-type disease resistance genes were identified in this *PMR1* region. Genotyping-by-sequencing (GBS) and genetic mapping analysis revealed suppressed recombination in the *PMR1* region, perhaps due to alien introgression. In addition, a comparison of species-specific InDel markers as well as GBS-derived SNP markers indicated that *C. baccatum* represents a possible source of such alien introgression of powdery mildew resistance into ‘VK515R'. The molecular markers developed in this study will be especially helpful for marker-assisted selection in pepper breeding programs for powdery mildew resistance.

## Introduction

*Leveillula taurica*, an obligate fungal plant pathogen belonging to the ascomycetes, causes powdery mildew in various vegetable crops, resulting in significant quality and yield losses. In the past few decades, the incidence of *L. taurica* powdery mildew has been increasing in both greenhouse- and open field-grown peppers worldwide (Damicone, 2009; Sudha and Lakshmanan, 2009; Cerkauskas et al., 2011). Premature defoliation caused by powdery mildew severely reduces crop yields and makes fruits unfavorable for marketing. Unlike most fungi that cause powdery mildew, which are epiphytic, *L. taurica* is an endophytic fungus, which hinders the efficacy of chemical control (Elad et al., 2007). Therefore, developing powdery mildew disease resistance in pepper is one of the main objectives of genetic and breeding programs.

Introgressing disease resistance from wild species into popular cultivars greatly increases crop yields and quality. Resistance sources for various pepper diseases have been reported in several wild species, and resistance has been successfully introgressed into commercial pepper cultivars, including resistance to tobamoviruses from *Capsicum chacoense* and *C. chinense* (Boukema, 1980; Berzal-Herranz et al., 1995; de la Cruz et al., 1997), resistance to tomato spotted wilt virus (TSWV) from *C. chinense* and *C. baccatum* (Boiteux et al., 1993; Hoang et al., 2013; Soler et al., 2015), resistance to anthracnose fruit rot from *C. chinense* (Voorrips et al., 2004), resistance to *Phytophthora capsici* from *C. annuum* cv. CM334 (Mallard et al., 2013; Liu et al., 2014) and resistance to bacterial leaf spot disease from *C. annuum* and *C. chacoense* (Cook and Guevara, 1984; Kim and Hartmann, 1985; Hibberd et al., 1987; Vallejos et al., 2010).

Pepper genotypes showing varied resistance levels against powdery mildew have been identified in *C. annuum, C. frutescens, C. baccatum*, and *C. chinense* (Ullassa et al., 1981; Deshpande et al., 1985; Pochard et al., 1986; Anand et al., 1987; De Souza and Café-Filho, 2003). Pepper genotypes ‘H-V-12' and ‘4638' (*C. annuum*), ‘IHR 703' (*C. frutescens*), and CNPH 36, 38, 50, 52, 279, and 288 (*C. baccatum*) are resistant to *L. taurica* (Anand et al., 1987; De Souza and Café-Filho, 2003). According to De Souza and Café-Filho (2003), most *C. annuum* species are moderately to highly susceptible to powdery mildew, whereas *C. baccatum, C. chinense*, and *C. frutescens* species are often resistant, suggesting that among *Capsicum* species, resistance to powdery mildew is primarily found in taxa other than *C. annuum*.

Powdery mildew resistance in pepper is reported to be a dominant and polygenic trait (Anand et al., 1987; Murthy and Deshpande, 1997; Blat et al., 2005). Genetic analyses have also indicated that relatively few genetic factors with significant additive and epistatic effects confer resistance to powdery mildew in different pepper genetic backgrounds (Daubèze et al., 1995; Murthy and Deshpande, 1997; Blat et al., 2005, 2006). At least three pairs of incompletely dominant genes are thought to confer resistance to powdery mildew in the *C. frutescens* line ‘IHR 703' (Anand et al., 1987). The most important and durable source of powdery mildew resistance reported is in the small-fruited pungent *C. annuum* accession ‘H3' from Ethiopia (Daubèze et al., 1995; Lefebvre et al., 2003). In addition, the *L. taurica*-resistant Israeli pepper line ‘H-V-12' was derived from a cross between the resistant cultivar ‘H3' and the susceptible cultivar ‘Vania' (Shifriss et al., 1992); at least three genes appear to control resistance to *L. taurica* in ‘H3' (Shifriss et al., 1992; Daubèze et al., 1995). Lefebvre et al. (2003) identified seven genomic regions, including additive quantitative trait loci (QTLs) and epistatic loci, that contribute to the resistance of the cultivar ‘H3' using a double-haploid population derived from a cross between ‘H3' and “Vania.”

In the present study, we identified and mapped a novel powdery mildew resistance locus, *PMR1*, to pepper chromosome 4 using two segregating populations: 102 ‘VK515' F_2:3_ families derived from a cross between resistant parental line ‘VK515R' and susceptible parental line ‘VK515S' and 80 ‘PM Singang' F_2_ lines derived from the F_1_ ‘PM Singang' commercial hybrid. Based on genetic analysis, the *PMR1* locus was localized to 1.0 and 5.1 cM genetic intervals on lower arm of chromosome 4 in ‘VK515R' and ‘PM Singang', respectively. In addition, we performed InDel marker sequence comparisons and GBS analyses to infer the origin of *PMR1*. Identification of molecular markers linked to the *PMR1* locus in pepper in the present study should facilitate marker-assisted selection (MAS) in pepper breeding programs aimed at introgressing the powdery mildew disease resistance trait.

## Materials and methods

### Plant materials

*C. annuum* ‘VK515R' and ‘VK515S' are powdery mildew-resistant and susceptible parental lines, respectively, kindly provided by In-Tae Kim (Samsung Seeds Co., Ltd., Korea). *C. annuum* ‘PM Singang' is a powdery mildew resistant commercial F_1_ hybrid cultivar (Nongwoo Bio Co., Ltd., Korea). *C. annuum* ‘Bukang' is a susceptible commercial cultivar (Hungnong Seed Co., Korea). ‘VK515' families and the ‘PM Singang' F_2_ population were used to map the *PMR1* locus. ‘VK515' F_1_ plants were derived from a cross between ‘VK515R' and ‘VK515S' lines. The ‘VK515' and ‘PM Singang' F_1_ hybrids were self-pollinated to produce F_2_ seeds in a greenhouse in 2012 and 2014, respectively. ‘VK515' F_2_ plants were grown in separate pots without selection and harvested individually to produce F_2:3_ families in 2015. 80 ‘PM Singang' F_2_ individuals and 102 ‘VK515' F_2:3_ families with 20 individuals per family were used for subsequent disease evaluation and mapping.

### Inoculum preparation and disease infection

For inoculum preparation, white powdery mildew spores collected from naturally-infected plants were used to inoculate the abaxial sides of leaves via the dropping method (Kim et al., 2009). Powdery mildew-infected ‘Bukang' and ‘VK515S' plants were maintained in greenhouses (Seoul National University, Suwon, Korea) and used as a source of *L. taurica*. At the time of sowing of ‘VK515' F_2:3_ families in plastic trays (50 cell trays), infectious ‘Bukang' and ‘VK515S' plants were introduced at regular intervals. In the case of the ‘PM Singang' F_2_ population, plants were raised in 20 cm diameter plastic pots and evaluated for resistance to powdery mildew under natural infection conditions (Korea Research Institute of Chemical Technology, Daejeon, Korea).

### Disease evaluation

‘VK515' F_2:3_ families and ‘PM Singang' F_2_ plants were evaluated for powdery mildew disease resistance 60 days after sowing. ‘VK515R' and ‘PM Singang' served as the resistant controls, whereas ‘VK515S' and ‘Bukang' served as the susceptible controls. In ‘VK515' F_2:3_ families, the presence or absence of white fungal hyphae on infected leaves at 60 days after sowing was used as a measure of disease infection. For ‘PM Singang' F_2_ plants, intensity of white fungal hyphae observed on infected leaves was visually scored using following disease scale: 1 = no sign of disease; 2 = minute necrotic lesions with no detectable sporulation; 3 = few large sporulating lesions; 4 = numerous large sporulating lesions.

### Genomic DNA extraction

Total genomic DNA (gDNA) was extracted from young leaf tissue using the cetyltrimethylammonium bromide (CTAB) method (Doyle and Doyle, 1987). The quality and quantity of the gDNA were analyzed using a NanoDrop 2000 spectrophotometer (Thermo Scientific, Waltham, MA, USA).

### Chromosomal localization

Four hundred twelve previously reported SNP markers (Kang et al., 2014) were used to detect polymorphism between ‘VK515R' and ‘VK515S'. A total of 96 SNP markers with uniform distribution in 12 pepper chromosomes and showing clear polymorphism between ‘VK515R' and ‘VK515S' were selected for the localization study (Table S1). A total of 92 plants were randomly selected from the ‘VK515' 102 F_2_ population and used for the SNP genotyping assay. Genotyping was performed with the Fluidigm® EP1™ system according to the manufacturer's protocol (Fluidigm, San Francisco, CA, USA). Briefly, specific target amplification (STA) was performed in a 5 μL reaction containing 60 ng of the gDNA according to the manufacturer's recommendations (Wang et al., 2009). PCR cycling conditions were as follows: 95°C for 15 min, followed by 14 cycles of amplification consisting of 95°C for 15 s and 60°C for 2 min. SNPtype assays were carried out using STA products according to the manufacturer's protocol (Fluidigm, San Francisco, CA, USA). PCR thermal cycling conditions were as follows: 95°C for 15 s, 64°C for 45 s and 72°C for 15 s with a touchdown of −1°C per cycle from 64 to 61°C, followed by 34 cycles of 95°C for 15 s, 60°C for 45 s and 72°C for 15 s. Genotyping data were automatically generated from the end-point image of the genotyping chip using the Fluidigm SNP Genotyping Analysis tool.

### Marker development

Based on our initial chromosomal localization study, SNP marker KS16052G01 was found to be linked to the powdery mildew resistance trait. To develop additional markers, PCR primers (Tables S2, S3) were designed in a 3.0 cM region around marker KS16052G01 using genomic information for *Capsicum* (Kim et al., 2014; Qin et al., 2014). To detect polymorphism between the parents, direct sequencing of gDNA PCR products was carried out. PCR analyses were performed according to Liu et al. (2016). The PCR products were gel eluted and sequenced at the National Instrumentation Center for Environmental Management (NICEM), Seoul National University, Seoul, Korea. Polymorphic PCR bands were used to develop SCAR markers, and SNPs were used to develop cleaved amplified polymorphic sequence (CAPS) or high-resolution melt (HRM) markers. CAPS markers were designed using CAPS Designer (https://solgenomics.net/tools/caps_designer/caps_input.pl), and HRM markers were designed according to Park et al. (2009). A total of ten markers including seven HRM, two SCAR and one CAPS markers were used for mapping the *PMR1* locus (Table 1).

**Table 1:** Molecular markers linked to the powdery resistance gene *PMR1*.

**Marker**	**Type**	**Primer sequence**	**Chromosomal position**	**Amplicon (bp)**
ZL1_10691	SCAR	F: TCCTGTTTTCTCCCCCTTTT	210,691,582	1,160
		R: CTTTGGCAATATCCCGTTCA	210,692,741	1,700, 2,100
HZ2_11079B	HRM	F: CTCTTTCGTTTGTTTTGCTTCA	211,079,744	208
		R: CTTTCAGCTCCTCTCCCAGC	211,079,951	
CZ2_11628	CAPS	F: GCTAGGATCCTGCTCGTGAGA	211,628,651	166, 174
		R: GTTGCTCTTGCTTCTGCTGC	211,628,988	340
HZ1_11658	HRM	F: TGCAAAATTTGATTCTTATAGTGGG	211,658,439	114
		R: CCTGTCGAAACTACGAGTCAAAA	211,658,552	
ZL1_1826	SCAR	F: CGAAGTCATTAAAGTTCATTGGG	211,826,850	1,259
		R: GCAATAAATGCCCTTCCACA	211,827,919	1,070
HPGV_1313	HRM	F: GGGTTTTCACTCCTCTTTTGC	213,138,139	187
		R: TCCACCATGAAGGTGTAACG	213,138,325	
HPGV_1344	HRM	F: AAAAGGCAAGAGCATTACATGA	213,443,490	196
		R: TTGTTGTTGTCGTTGTTGTTGA	213,443,685	
HPGV_1412	HRM	F: TCTCGGAGGGAAAACTGAAA	214,125,960	178
		R: AAGCATAAGGGCATGTTTGG	214,126,137	
HRM4.1.6	HRM	F: AATTAAAGGACTTAAGTTTGACAGTT	215,120,006	203
		R: GAAATTGTCGATGAACATCCGT	215,119,805	
HRM2_A4	HRM	F: TTCAGCCAGTGATCTGGAGC	215,542,952	169
		R: TCAAATTCCTTGCACAAATCAT	215,543,120	

### Genotyping and molecular mapping

A total of 102 ‘VK515' F_2_ individuals, 102 ‘VK515' F_2:3_ families, and 80 ‘PM Singang' F_2_ individuals were used to map the *PMR1* locus. For genotyping with SCAR markers, PCR was performed in 25 μL reactions, including 2.5 μL 10 × buffer, 2 μL 10 mM dNTPs, 1 μL 10 μM each of forward and reverse primer, 15.2 μL distilled H_2_O, 0.3 μL Taq DNA polymerase, and 3 μL of 20 ng/μL gDNA. The PCR program was 95°C for 5 min, 35 cycles of 95°C for 30 s, 56°C for 30 s, and 72°C for 60 s, followed by a final extension of 72°C for 10 min. For genotyping with CAPS marker (CZ2_11628), PCR was performed as mentioned above, but with an extension time of 72°C for 30 s. Amplified PCR products were digested with the restriction enzyme *Taq*^α^I according to the manufacturer's protocol (New England Biolabs, Beverly, MA). Digested PCR products were separated by gel electrophoresis and observed under a UV transilluminator (Bio-Rad, USA).

HRM analysis was carried out using a LightScanner® (Idaho Technology Inc., USA). For HRM analysis, PCR was performed in 20 μL of reaction mixture, including 2 μL 10 × buffer, 0.25 mM dNTPs, 5 pmol primer mix, 1 unit Taq polymerase, 1.25 μM Syto9, and 50 ng gDNA. The PCR program was 95°C for 5 min, 35 cycles of 95°C for 30 s, 56°C for 30 s, and 72°C for 30 s, followed by a final extension of 72°C for 10 min. HRM was run using 0.1°C increments between 70 and 90°C, with each increment held for 1 s.

Linkage analysis was performed using CarthaGene Software (Schiex and Gaspin, 1997). To construct a linkage group, the LOD threshold was set at 5.0, and the maximum distance was set at 30 cM. Members of linkage groups were determined based on the physical positions of the markers in ‘L_Zunla-1'. Genetic distances between markers were determined in cM using the Kosambi mapping function. The resulting genetic linkage map was drawn using MapChart 2.3 software (Voorrips, 2002).

### Sequence comparison of *PMR1* locus-specific indel markers

To compare the *PMR1* locus-specific InDel markers, the sequences of two *PMR1* locus-specific InDel markers (ZL1_1826 and Chr4.1.6) from *C. annuum* ‘VK515' parents were PCR amplified using ZL1_1826 and Chr4.1.6 primer sets (Tables S2, S3) as described above and sequenced at NICEM, Seoul National University, Seoul, Korea. The *PMR1* locus-specific InDel markers sequences from *C. annuum* ‘L_Zunla-1' (http://peppersequence.genomics.cn), *C. chinense* ‘PI159236' (version 1.2), and *C. baccatum* ‘PBC81' (version 1.2) (http://peppergenome.snu.ac.kr/download.php) were retrieved from corresponding genome databases (Kim et al., 2017). InDel marker sequences were aligned using the MAFFT multiple sequence alignment program (http://www.ebi.ac.uk/Tools/msa/mafft/). The sequence alignments were illustrated using the Jalview tool (Waterhouse et al., 2009).

### Prediction of *PMR1* candidate genes

Gene predictions were performed with the FGENESH tool (Solovyev et al., 2006). The predicted protein sequences from the *PMR1* region were BLASTP searched against the NCBI database for disease resistance-related genes. Protein domain analysis of the candidate resistance (*R*) genes from the *PMR1* locus was carried out at te Pfam database (http://pfam.xfam.org/).

### Genotyping-by-sequencing (GBS)

GBS was performed using 12 ‘VK515' F_2_ individuals, including 11 homozygous resistant genotypes and one heterozygous genotype, along with two replications of parents ‘VK515R' and ‘VK515S'. To construct comprehensive and representative libraries for Illumina sequencing, 400 ng of gDNA was used. Libraries for GBS were constructed as described by Truong et al. (2012). Briefly, gDNA from the parental lines (‘VK515R' and ‘VK515S') and 12 F_2_ lines was digested with *Pst*I and *Mse*I. *Mse*I adapters and the *Pst*I adapter with different barcodes for each sample were ligated to the digested gDNA fragments. After amplification, the quality and quantity of the libraries were evaluated using a Bioanalyzer DNA 1000 Chip (Agilent Technologies, Santa Clara, CA, USA). The same amount of adapter-ligated fragments from each sample was pooled for sequencing.

Sequencing was performed at Macrogen (Seoul, Korea) using a HiSeq 2000 (Illumina, San Diego, CA, USA). Raw reads were demultiplexed in accordance with individual barcodes, and the adapter and barcode sequences were removed using commercially available CLC genomics workbench software version 8.0 (CLC Bio, Aarhus, Denmark). Trimmed reads were mapped to genomic sequences of *C. annuum* ‘L_Zunla-1' version 2.0 (Qin et al., 2014; http://peppersequence.genomics.cn) using Burrows-Wheeler Aligner (BWA) version 0.7.12 (Li, 2013). Picard Tools version 1.119 and SAMtools version 1.1 were used for read grouping and sorting (Li et al., 2009). For genome-wide SNP calling, Genome Analysis Toolkit (GATK) UnifiedGenotyper version 3.3 was used. High-quality SNPs with QUAL value larger than 30 and minimum depth 3 were selected for further analysis.

The sequence regions corresponding to the *PMR1* locus from *C. annuum* ‘L_Zunla-1', *C. chinense*, and *C. baccatum* chromosome sequences were aligned with GBS-SNP data from the ‘VK515' genome. Phylogenetic analysis was carried out using DARwin 6.0.9 (Perrier and Jacquemoud-Collet, 2006).

## Results

### Inheritance analysis of powdery mildew resistance

**Figure 1:**
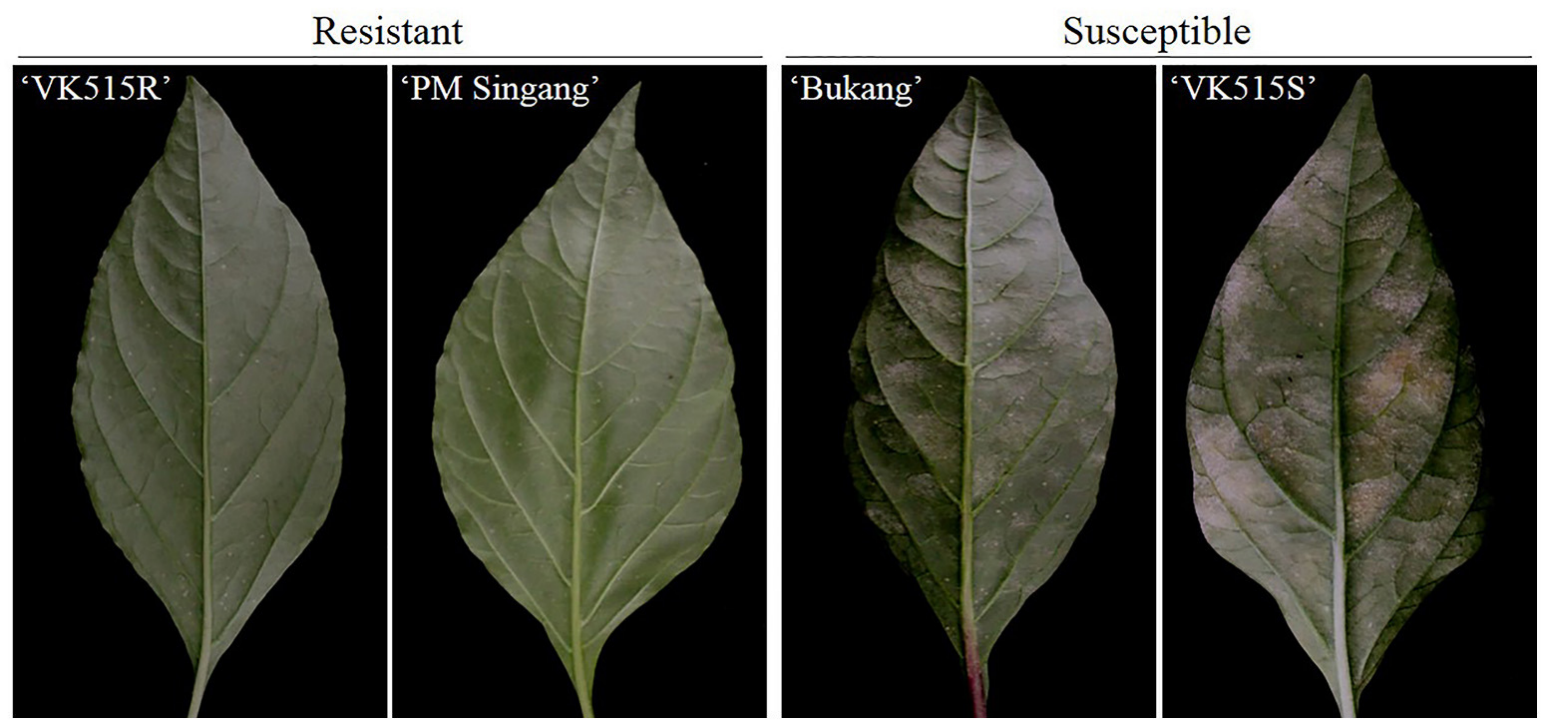
Phenotypic analysis of powdery mildew resistance in pepper. Comparison of the phenotypes of resistant (‘VK515R') and susceptible (‘VK515S') parental lines and resistant (‘PM Singang') and susceptible (‘Bukang') commercial pepper cultivars infected with *L. taurica*.

We evaluated ‘VK515' F_2:3_ families along with resistant (‘VK515R' and ‘PM Singang') and susceptible (‘VK515S' and ‘Bukang') pepper lines infected with *L. taurica* for powdery mildew disease resistance 60 days after infection. Powdery mildew infection was evident by the appearance of mycelial growth on the leaf surface of susceptible controls. Powdery mildew-resistant ‘PM Singang' and ‘VK515R' failed to show disease symptoms, as no white fungal hyphae were observed on the abaxial surfaces of leaves. Similar results were obtained for ‘VK515' F_1_ plants, whereas susceptible ‘Bukang' and ‘VK515S' plants showed white fungal hyphae on the abaxial sides of leaves (Figure 1). Among the 102 ‘VK515' F_2:3_ families, 24 families (total of 451 plants) were homozygous resistant, 48 families (total of 898 plants) were segregating for this trait, and 30 families (total of 582 plants) were homozygous susceptible, which showed a good fit to a 1:2:1 ratio (χ^2^ = 1.06; *P* = 0.59) (Table 2). In the case of the ‘PM Singang' F_2_ population, plants displaying a disease score value of one or two were considered as resistant, whereas plants scored as three or four were considered as susceptible (Figure S1). Out of 80 ‘PM Singang' F_2_ lines, 59 plants were resistant and 21 plants were susceptible, which fit to a 3:1 ratio (χ^2^ = 0.02; *P* = 0.80) (Table S4). Overall, these results suggest that resistance to powdery mildew is controlled by a single dominant locus, *PMR1*, in these pepper populations.

**Table 2:** Segregation analysis of powdery mildew resistance in ‘VK515' families.

**Population**	**Number of plants/families**	**Phenotype**			**Expected ratio**	**χ^2^**	***P*-value**
		**R**	**H**	**S**			
‘VK515R'	20	20					
‘VK515S'	20			20			
‘VK515' F_1_	20	20					
‘VK515' F_2:3_ families	102 (1,931)	24 (451)	48 (898)	30 (582)	1:2:1	1.06	0.59

*R, resistant; H, heterozygote; S, susceptible; Number of F_2:3_ ‘VK515' plants used are indicated in parentheses*.

### Marker development and genetic mapping of the *PMR1* locus

**Figure 2:**
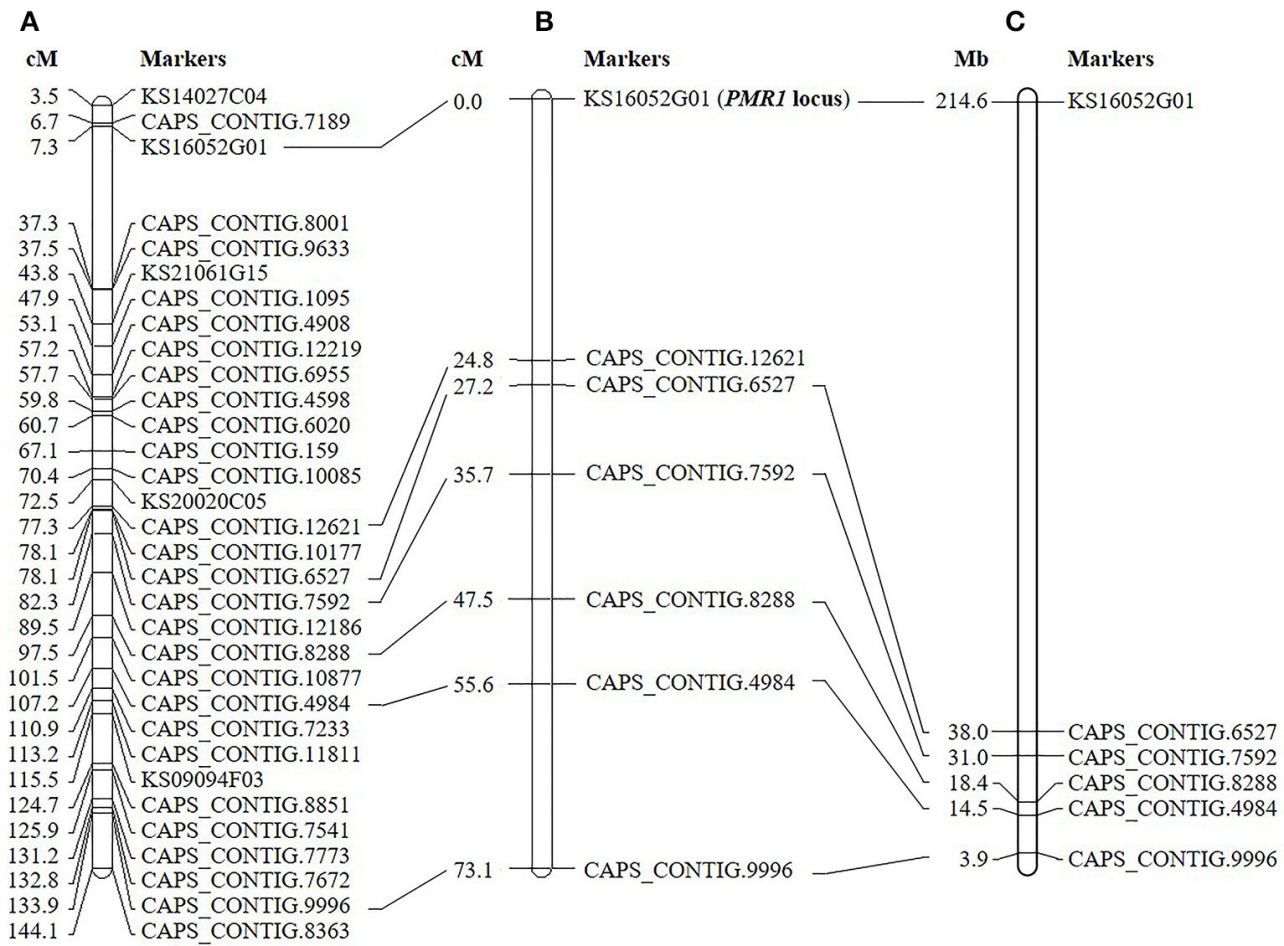
Linkage map of the pepper *PMR1* locus. **(A)** Genetic map of *C. annuum* reported by Kang et al. (2014). **(B)** Seven markers linked to the *PMR1* locus based on the ‘VK515' F_2:3_ mapping population are shown. **(C)** Physical locations of SNP markers on ‘L_Zunla-1' chromosome 4.

Initial genotyping and linkage analysis of ‘VK515' F_2_ population with 96 SNP markers (Kang et al., 2014) identified seven SNP markers (KS16052G01, CAPS_CONTIG.12621, CAPS_CONTIG.6527, CAPS_CONTIG.7592, CAPS_CONTIG.8288, CAPS_CONTIG.4984, and CAPS_CONTIG.9996) linked to the *PMR1* locus on linkage group 4 (Figure 2A). The closest markers, KS16052G01 and CAPS_CONTIG.12621 were 0 and 24.8 cM away from the *PMR1* locus, respectively (Figure 2B). The physical interval between KS16052G01 and CAPS_CONTING.6527 markers was approximately 176.6 Mb on chromosome 4 of ‘L_Zunla-1' (Figure 2C). Therefore, more markers were required to saturate the *PMR1* locus.

**Figure 3:**
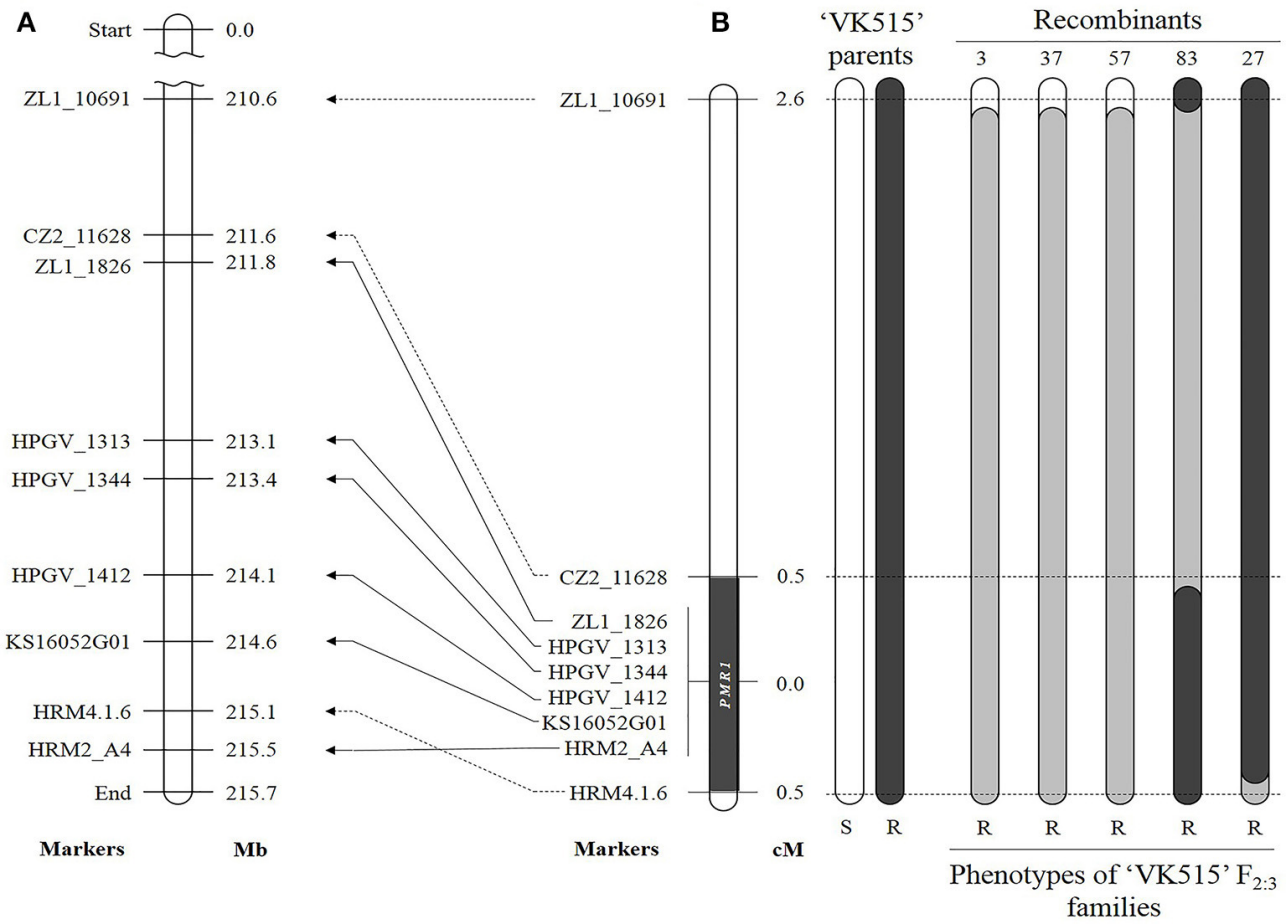
Comparative genetic linkage and physical maps of the powdery mildew resistance gene *PMR1*. **(A)** Physical locations of linked markers on ‘L_Zunla-1' chromosome 4. **(B)** Genetic map of ‘VK515' F_2:3_ families. Recombinant heterozygous resistant plants 3, 37, and 57; homozygous resistant plants 27 and 83. Eight SNP markers linked to the *PMR1* locus are indicated next to pepper chromosome 4. Numbers on the right indicate genetic distances (cM). Black and white rectangles indicate the homozygous intervals of ‘VK515R' and ‘VK515S' chromosome 4, and gray rectangles indicate heterozygous intervals. The *PMR1* locus was delimited to a 4 Mb-region between CZ2_11628 and HRM4.1.6 on ‘L_Zunla-1' chromosome 4.

We developed additional markers linked to the *PMR1* locus using genomic information for *Capsicum*. To find SNPs by direct sequencing, we designed 12 and 26 primer sets around the *PMR1* region (176.6 Mb) on chromosome 4, respectively based on the ‘CM334' (Table S2) and ‘L_Zunla-1' (Table S3) genome sequence (Kim et al., 2014; Qin et al., 2014). Seven SNPs were identified through direct sequencing; one SNP with Chr4.1.6, two SNPs with each of the A4, ZL1_11079B, and ZL1_11658 primer sets. These SNPs were converted into four HRM markers, HRM4.1.6, HRM2_A4, HZ2_11079B, and HZ1_11658, respectively. Of these markers, HRM2_A4 and HRM4.1.6 showed polymorphism and co-segregated with the resistance phenotype in ‘VK515' population (Figure S2, Figure 3A). Additionally, we developed two SCAR markers, ZL1_1826 and ZL1_10691, and one CAPS marker, CZ2_11628 (Table 1, Figure S2) based on ‘L_Zunla-1' genome sequence information.

A total of six polymorphic markers (ZL1_10691, CZ2_11628, ZL1_1826, HRM2_A4, HRM4.1.6, and KS16052G01) were mapped in the 102 ‘VK515' F_2:3_ families (Table 1, Figure 3B). Based on genotyping analysis of the 102 ‘VK515' F_2:3_ families (1931 plants), three recombinants with ZL1_10691 and one recombinant for each of the CZ2_11628 and HRM4.1.6 markers were identified (Figure 3B). Based on genetic analysis, the *PMR1* locus was delimited to a 1.0 cM-region between markers CZ2_11628 and HRM4.1.6 on chromosome 4 (Figure 3B). We found that this 1 cM-region corresponded to a DNA fragment of an approximately 4 Mb-region in the lower arm of ‘L_Zunla-1' chromosome 4. The physical location of HRM4.1.6 showed no collinearity with the corresponding position in ‘L_Zunla-1' (Figure 3), perhaps due to mis-assembly of the contig sequence. Among the six markers used, three markers, ZL1_1826, KS16052G01, and HRM2_A4, co-segregated with the powdery mildew resistance phenotype and were found to be at a genetic distance of 0 cM from the *PMR1* locus (Figure 3B).

For ‘PM Singang', seven molecular markers, which were found to be polymorphic (ZL1_10691, HZ2_11079B, CZ2_11628, HZ1_11658, ZL1_1826, HRM2_A4, and HRM4.1.6) were used to map the *PMR1* locus (Table 1, Figure S3). The *PMR1* locus was mapped in 5.1 cM interval flanked by HZ2_11079B and HRM2_A4. Marker order was found to be identical to that of ‘VK515', with the exception of markers HZ2_11079B and HZ1_11658, which were not polymorphic in the ‘VK515' population. The total genetic lengths covered by the flanking markers in the ‘VK515' and ‘PM Singang' population were 3.1 and 8.9 cM, respectively (Figure S3).

### Investigating the origin of the *PMR1* locus

**Figure 4:**
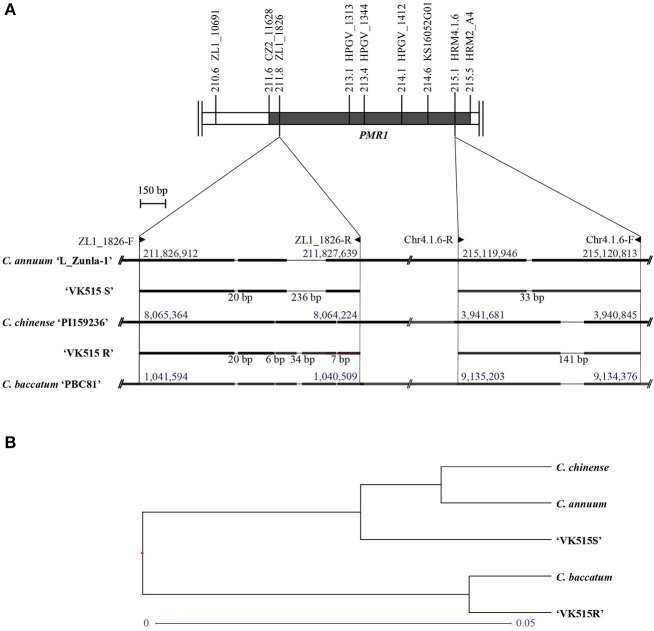
Sequence analysis of the *PMR1* locus. **(A)** Schematic representation of ‘L_Zunla-1', *C. chinense*, and *C. baccatum PMR1*-specific InDels and corresponding sequences of ‘VK515' parental lines. InDel lengths in bp are shown below the solid lines. Numbers (blue font) above blocks indicate location on chromosome 4. Primer positions of the *PMR1* linked markers are indicated with triangles. **(B)** Phylogenetic analysis of the *PMR1* locus based on GBS data.

To investigate the origin of the *PMR1* locus, we performed sequence comparisons of the InDel markers (ZL1_1826 and Chr4.1.6) among *Capsicum* species. The ZL1_1826 and Chr4.1.6 marker sequences from ‘VK515R' and ‘VK515S' were aligned with corresponding sequences from *C. annuum* ‘L_Zunla-1', *C. chinense*, and *C. baccatum* (Figure 4, Figure S4). Sequence alignment of ZL1_1826 revealed an InDel of 236 bp that was specific to *C. annuum* ‘L_Zunla-1' and ‘VK515S'; a 34 bp InDel was found only in the ‘VK515R' and *C. baccatum* genomes. A 20 bp InDel was found in all species except *C. chinense* (Figure 4A, Figure S4), whereas 6 and 7 bp InDels were specific to *C. chinense, C. baccatum*, and ‘VK515R'. The Chr4.1.6 marker showed a 141 bp InDel specific to *C. annuum* ‘VK515R', *C. chinense*, and *C. baccatum*, whereas *C. annuum* ‘VK515S' and ‘L_Zunla-1' showed a 33 bp InDel. The presence of ZL1_1826 and Chr4.1.6 marker sequences with species-specific InDels indicated that the *PMR1* locus from ‘VK515R' was more closely related to those of *C. baccatum* and *C. chinense* than those of ‘VK515S' and *C. annuum*, suggesting that the *PMR1* region in ‘VK515R' may have resulted from an alien introgression of chromosomal segments from either *C. chinense* or *C. baccatum* (Figure 4A, Figure S4).

To further confirm the origin of the *PMR1* locus, as well as the suppressed recombination in the *PMR1* locus, we aligned the GBS data with genomic sequences from *C. annuum* ‘L_Zunla-1', *C. chinense*, and *C. baccatum*. Based on GBS data analysis, 22 SNPs (Table S5) were detected in the *PMR1* locus, nine of which were converted into three HRM markers, HPGV_1313 (SNP4, SNP5, and SNP6), HPGV_1344 (SNP7), and HPGV_1412 (SNP8, SNP9, SNP10, SNP11, and SNP12) for genotyping. However, no recombinants were found with these markers, further confirming the suppressed recombination in the *PMR1* region. Furthermore, phylogenetic analysis of the GBS data (Figure 4B) from the *PMR1* locus revealed clustering of ‘VK515S', *C. annuum*, and *C. chinense*, whereas ‘VK515R' and *C. baccatum* shared a common node, suggesting that the *PMR1* locus in ‘VK515R' is closely related to that of *C. baccatum*. These results suggest that the *PMR1* locus might have been introgressed from *C. baccatum*.

## Discussion

In the present study, we investigated the genetic factor responsible for powdery mildew resistance in two independent sources of powdery mildew resistance, *C. annuum* ‘VK515R' and ‘PM Singang'. Genetic analysis confirmed that a single dominant locus, *PMR1*, which mapped to chromosome 4 of the pepper genome, is responsible for the powdery mildew resistance in these cultivars. Synteny analysis between the pepper *PMR1* locus and the tomato genome showed that much of the *PMR1*-region is syntenic with tomato chromosome 3 (Figure S5). The *PMR1* locus was mapped to a syntenic region in two independent pepper populations, ‘VK515' and ‘PM Singang'. The genetic distance between the flanking markers CZ2_11628 and HRM4.1.6 in ‘VK515' F_2:3_ families and HZ2_11079B and HRM2_A4 in the ‘PM Singang' population were 1.0 and 5.1 cM with total map distances of 3.1 and 8.9 cM, respectively. The difference in genetic distance between the ‘VK515' and ‘PM Singang' populations is likely due to the difference in the size and nature of the genetic populations (having 102 F_2:3_ families vs. 80 F_2_ individuals).

Previously, Lefebvre et al. (2003) mapped powdery mildew resistance QTLs in a double-haploid population derived from a cross between ‘H3' (resistant) and ‘Vania' (susceptible). Five additive QTLs (located on chromosomal regions P5, P6, P9, P10, and P12) and two epistatic interactions together explaining more than 50% of the genotypic variance were detected (Lefebvre et al., 2003). Eggink et al. (2016) reported a QTL on LG1/8 explaining about 57% phenotypic variance from an unknown pepper population in their patent application. However, none of these correspond to the dominant *PMR1* locus identified in this study. Recently, Gabor et al. (2017) reported a powdery mildew-resistance QTL located on chromosome 4 (in an interval of about < 40 cM between markers NE0235653 and NE0240958) in pepper line PBC167. Thus, previous genetic analyses indicate a complex mode of inheritance for powdery mildew resistance in pepper. Genetic analyses have also indicated dominant inheritance of powdery mildew resistance in certain pepper backgrounds (Anand et al., 1987). The dominant resistance sources in ‘VK515' and ‘PM Singang' are different from other known powdery mildew resistance factors reported. Overall, previous findings and the results presented here indicate that the nature of genetic resistance to powdery mildew in pepper genotypes is highly variable, and it is likely that different genes are involved in conferring the powdery mildew resistance in different genetic backgrounds (Daubèze et al., 1995; Murthy and Deshpande, 1997; Lefebvre et al., 2003; Blat et al., 2005, 2006).

Several studies have been carried out to identify and analyze the powdery mildew disease resistance trait in other crops. In particular, powdery mildew resistance conferred by the loss-of-function alleles of specific *MLO* (*Mildew Locus O*) genes due to recessive mutations, such as in *Arabidopsis* (*AtMLO2*), barley (*Mlo*), and tomato (*SlMlo1*) and their mutants have been utilized in breeding programs to provide effective *mlo*-mediated powdery mildew resistance (Büschges et al., 1997; Bai et al., 2008; Pavan et al., 2011; Zheng et al., 2013). *MLO* genes encode proteins with seven transmembrane domains and have a recessive inheritance pattern (Pessina et al., 2014). Unlike *MLO* genes, *PMR1* showed dominant inheritance. Several dominant *R* genes, such as *RPW8* from *Arabidopsis* (Xiao et al., 2001), *Pm3b* from wheat (Yahiaoui et al., 2004; Srichumpa et al., 2005), and *Mla6, Mla1*, and *Mla13* from barley (Halterman et al., 2001, 2003; Zhou et al., 2001), belong to the NB-ARC domain-containing *R* gene family. In the present study, among the 622 predicted genes from the 4-Mb region of the *PMR1* locus, two genes (FGENESH: 408 and FGENESH: 556) were found to encode R proteins sharing sequence similarity with NBS-LRR domain-containing R proteins (Table S6). These genes represent potential candidates for pepper powdery mildew resistance genes. However, the possible role of leucine-rich repeat receptor kinases in plant powdery mildew resistance cannot be ruled out, as these proteins play crucial roles in a wide variety of plant developmental and defense-related processes, including both host-specific and non-host-specific defense responses (Torii, 2004), and several of the predicted genes from the pepper *PMR1* region were found to share sequence similarity with leucine-rich repeat receptor kinase genes.

The flanking markers CZ2_11628 and HRM4.1.6 delimited the *PMR1* locus in ‘VK515R' to an interval of 1.0 cM corresponding to a DNA fragment of an approximately 4-Mb region in the lower arm of ‘L_Zunla-1' chromosome 4. Suppressed recombination and segregation distortion are two common problems in populations segregating for alien introgressions, which can affect genetic mapping and linkage analyses (Chetelat et al., 2000; Neu et al., 2002; Pertuzé et al., 2003; Canady et al., 2006; Nagy et al., 2010). Consistent with this notion, the present analysis, which is based on genotyping as well as GBS analysis, indicated suppressed recombination between markers CZ2_11628 and HRM4.1.6, each spanning a genetic distance of 0.5 cM from the *PMR1* locus, which might have been introgressed from a wild *Capsicum* species. In accordance with this possible introgression, phylogenetic analysis of the GBS data from the *PMR1* locus showed that ‘VK515R' shared a common node with *C. baccatum*. Similar results were obtained with phylogenetic analysis of the *PMR1* locus-specific InDel marker sequences (data not shown). These results further substantiate the idea that the *PMR1* locus might have been introgressed from *C. baccatum*. Nevertheless, a possible role of chromosomal rearrangements in the suppressed recombination at the *PMR1* region cannot be ruled out (Neu et al., 2002).

Screening for powdery mildew resistance in numerous pepper genotypes (De Souza and Café-Filho, 2003) has suggested that resistance to powdery mildew most likely originated from species other than those of the *C. annuum* taxon. Furthermore, the dominant pattern of inheritance of powdery mildew resistance in ‘VK515R', which is similar to that of *C. baccatum* (Blat et al., 2005), supports the notion that powdery mildew resistance in ‘VK515R' might have introgressed from *C. baccatum*, possibly using *C. chinense* as a bridge species, since *C. annuum* and *C. baccatum* are not cross-compatible (Manzur et al., 2015; Martins et al., 2015). Indeed, phylogenetic analysis based on GBS data and InDel markers demonstrated the close relatedness of the *PMR1* region from *C. baccatum* and *C. annuum* ‘VK515R', further supporting the introgression of the *PMR1* locus from *C. baccatum*.

In summary, phenotypic and genetic analysis of powdery mildew resistance in pepper cultivars, VK515R and PM Singang identified a single dominant locus for powdery mildew resistance. We mapped the *PMR1* locus to 1.0 and 5.1 cM intervals on chromosome 4 of ‘VK515R' and ‘PM Singang'. Six and three molecular markers co-segregating with the powdery mildew resistance were identified from ‘VK515R' and ‘PM Singang', respectively. The molecular markers that we developed in this study will be especially useful for MAS and pyramiding of powdery mildew-resistance genes into elite cultivars, as they are tightly linked to the *PMR1* locus, and efficient SNP marker detection platforms are currently available. Further fine mapping and candidate gene analyses are needed to uncover the relationship between the predicted receptor kinase/NBS-LRR genes and *PMR1*.

## Author contributions

Conceived and designed the experiments: B-CK and JJ. Participated in phenotyping: JJ and GC. Participated in marker development and genetic mapping: JJ. Performed GBS analysis: JJ, KH, H-YL. Drafted the manuscript: JJ, JV. Revised the manuscript: JV, B-CK, HL, DC.

### Conflict of interest statement

The authors declare that the research was conducted in the absence of any commercial or financial relationships that could be construed as a potential conflict of interest.

## Supplementary material

Figure S1 Scoring of powdery mildew resistance in F_2_ ‘PM Singang' population. Disease score values 1, 2, 3, and 4 indicate no sign of disease development, minute necrotic lesions with no detectable sporulation, few large sporulating lesions, and numerous large sporulating lesions, respectively.

Figure S2 Analysis of molecular markers. **(A)** PCR analysis of SCAR marker ZL1_1826 and CAPS marker CZ2_11628. **(B)** Normalized HRM curves of SNP-based markers HRM4.1.6 and HRM2_A4. RR: genotype of resistant line, rr: genotype of susceptible line, Rr: genotype of heterozygous F_1_ plants.

Figure S3 Comparative map of the *PMR1* locus in two pepper populations, ‘VK515' and ‘PM Singang'.

Figure S4 Sequence comparisons of InDel markers flanking *PMR1*.

Figure S5 Syntenic relationship between pepper *PMR1* locus and tomato chromosome 4.

Table S1 96 SNP markers used in the Fluidigm genotyping assay.

Table S2 ‘CM334' genome-based primer sequences used for polymorphism detection.

Table S3 ‘L_Zunla-1' genome-based primer sequences used for polymorphism detection.

Table S4 Segregation analysis of powdery mildew resistance in ‘PM Singang' population.

Table S5 Genotyping-by-sequencing of the *PMR1* locus.

Table S6 Predicted genes from the *PMR1* region.
