# Supplementary figures and images for: Molecular Mapping of PMR1, a Novel Locus Conferring Resistance to Powdery Mildew in Pepper (Capsicum annuum)
Source: Front Plant Sci. 2017 Dec 8;8:2090. doi: 10.3389/fpls.2017.02090 (PMC5727091; doi:10.3389/fpls.2017.02090)

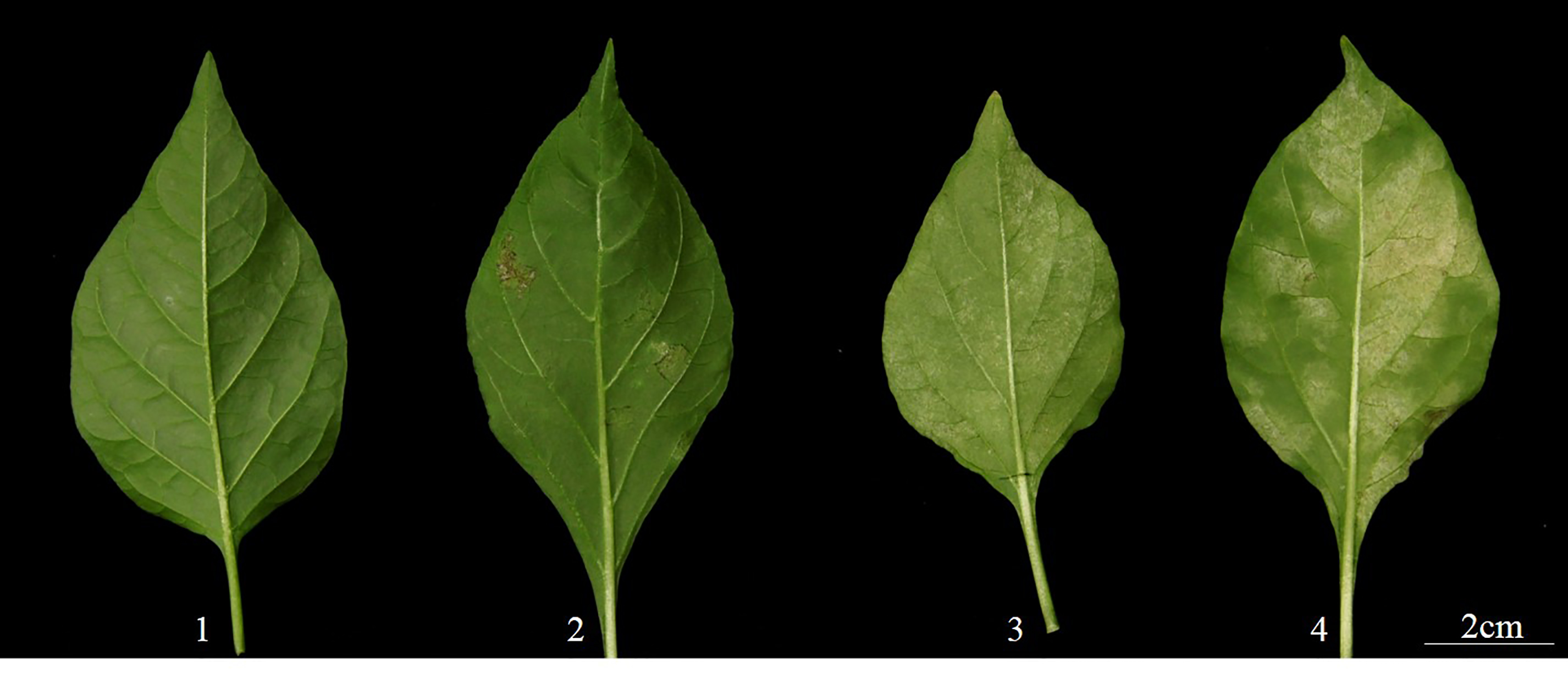

Supplement: Figure S1 — Scoring of powdery mildew resistance in F2 ‘PM Singang' population. Disease score values 1, 2, 3, and 4 indicate no sign of disease development, minute necrotic lesions with no detectable sporulation, few large sporulating lesions, and numerous large sporulating lesions, respectively. [file Image1.JPEG]

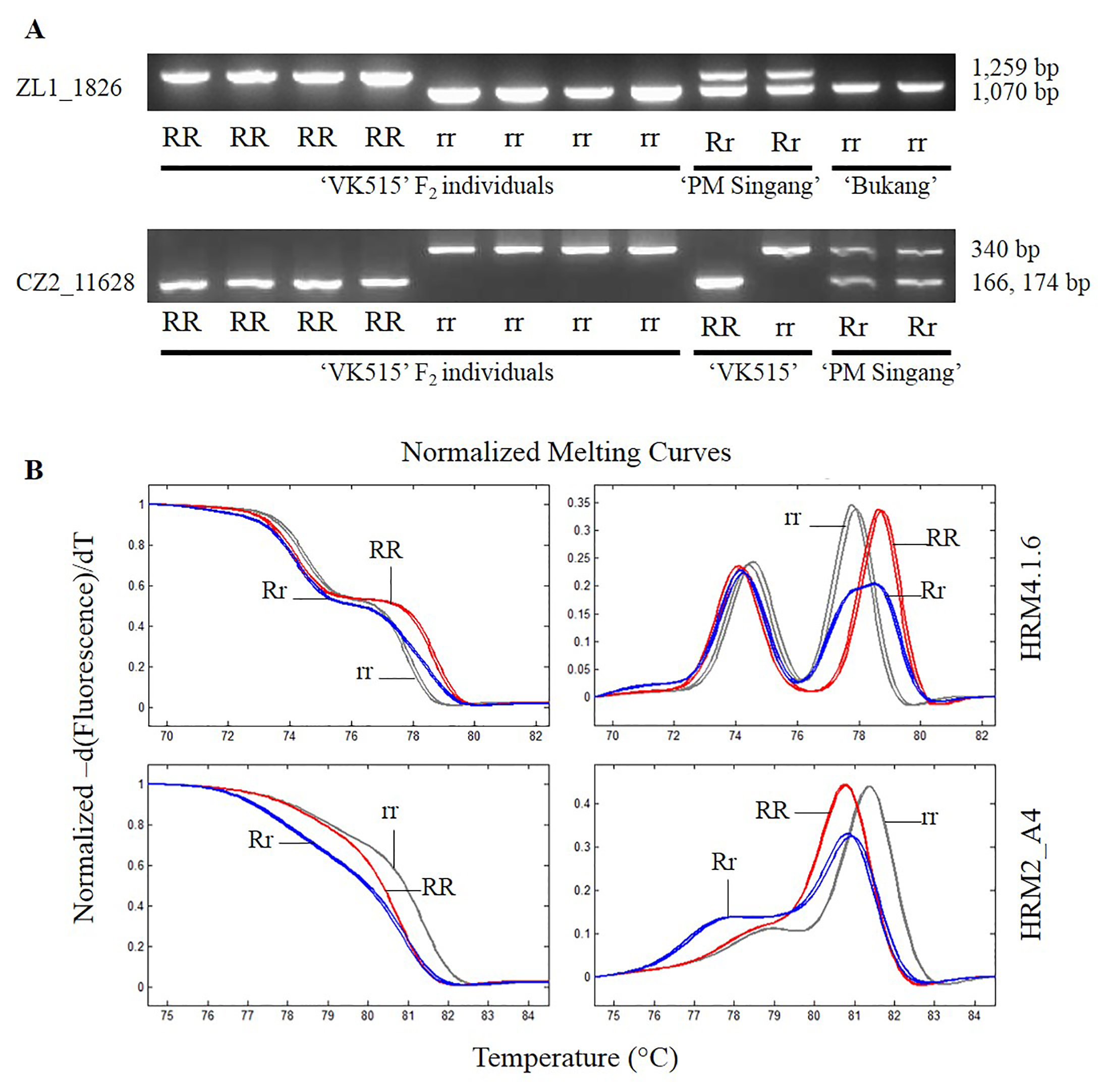

Supplement: Figure S2 — Analysis of molecular markers. (A) PCR analysis of SCAR marker ZL1_1826 and CAPS marker CZ2_11628. (B) Normalized HRM curves of SNP-based markers HRM4.1.6 and HRM2_A4. RR: genotype of resistant line, rr: genotype of susceptible line, Rr: genotype of heterozygous F1 plants. [file Image2.JPEG]

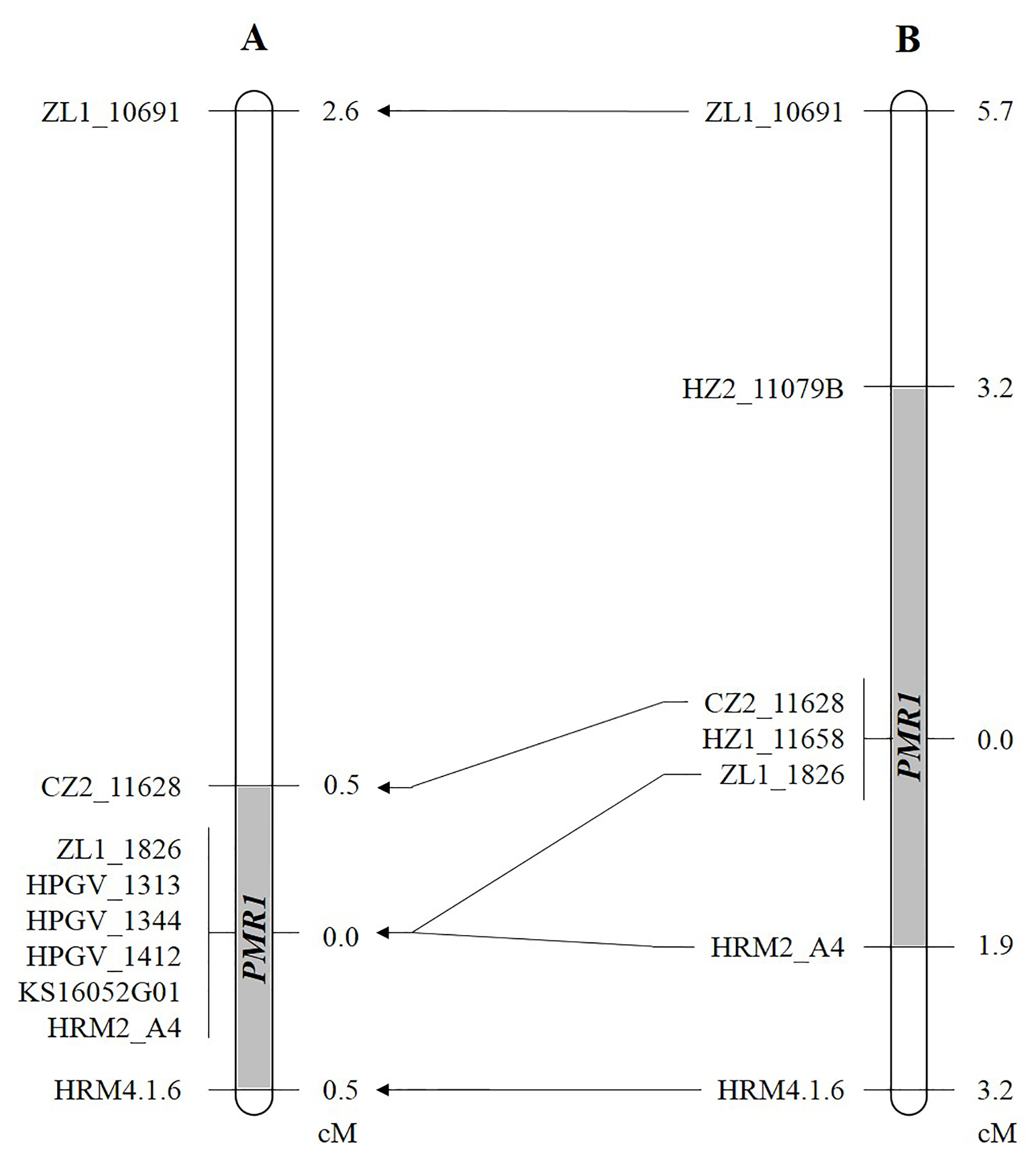

Supplement: Figure S3 — Comparative map of the PMR1 locus in two pepper populations, ‘VK515' and ‘PM Singang'. [file Image3.JPEG]

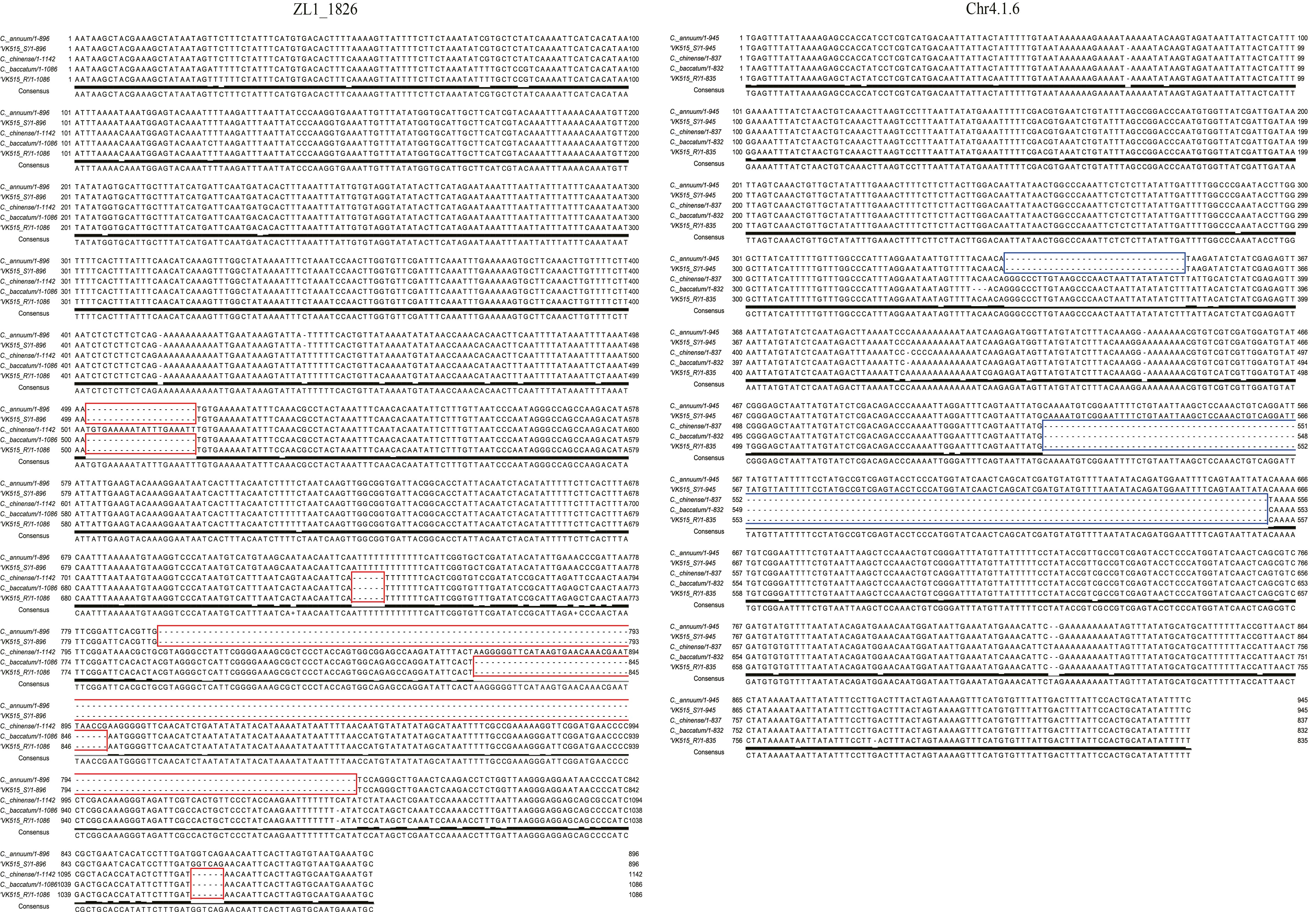

Supplement: Figure S4 — Sequence comparisons of InDel markers flanking PMR1. [file Image4.JPEG]

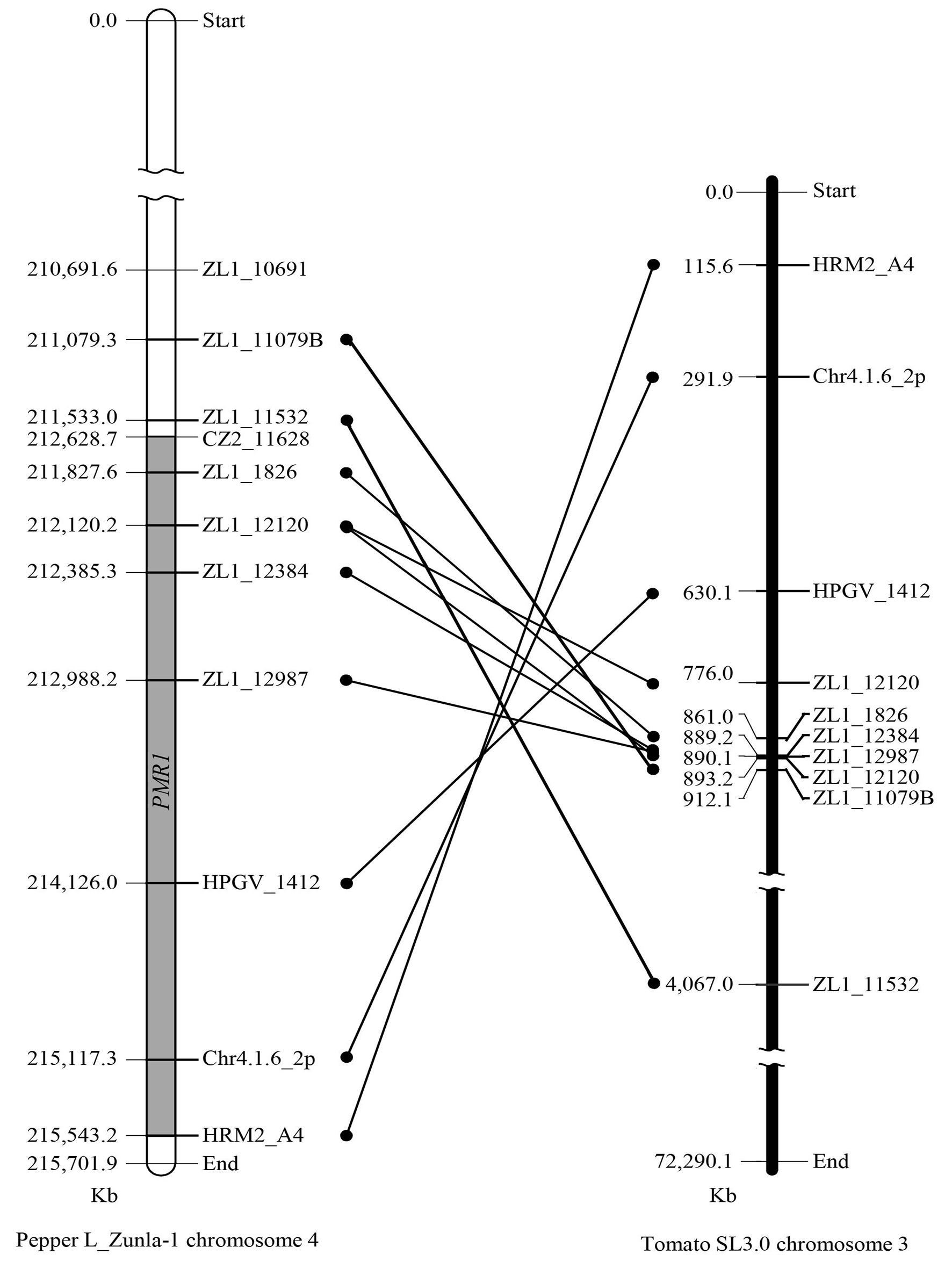

Supplement: Figure S5 — Syntenic relationship between pepper PMR1 locus and tomato chromosome 4. [file Image5.JPEG]
