# Supplementary material for: Molecular Mapping of PMR1, a Novel Locus Conferring Resistance to Powdery Mildew in Pepper (Capsicum annuum)
Source: Front Plant Sci. 2017 Dec 8;8:2090. doi: 10.3389/fpls.2017.02090 (PMC5727091; doi:10.3389/fpls.2017.02090)
Supplement: Table S2 — ‘CM334' genome-based primer sequences used for polymorphism detection. [file Table2.docx]

**Table S2 ‘**CM334’ genome-based primer sequences used for polymorphism detection

| **S.No** | **Primer** | **Sequence** | **Position on Chr4** | **Amplicon (bp)** |
| --- | --- | --- | --- | --- |
| 1 | 1H7 | F: TGGGCAGTCAGGAGAATCAA | 582 | 969 |
|  |  | R: AGCTCACAAACGGAGCAGTG | 1,550 |  |
| 2 | A4* | F: ACAGCTAGCGCTGAAAAGCA | 5,584 | 966 |
|  |  | R: TTCCGTGGCTTCTACACTGC | 6,549 |  |
| 3 | Ch4.1.5_3P | F: TGTTTGTATGGGCGGGTCTA | 406,496 | 884 |
|  |  | R: CAACTAAATGGCGCACCCTA | 407,379 |  |
| 4 | Ch4.1.5_19P | F: CGAAAGAAACAGCTCGAACG | 445,223 | 889 |
|  |  | R: CCAACACAAACGGGCAATAC | 446,111 |  |
| 5 | Ch4.1.5_39P | F: CAATCTGCGGGTAAATGTAAG | 498,219 | 947 |
|  |  | R: AAACATGGATTGAGCAGACTA | 499,165 |  |
| 6 | A2 | F: GGACACTTGGTCGCAATCTG | 778,836 | 802 |
|  |  | R: CTGGCTTTACGAGCGAGCTA | 778,035 |  |
| 7 | Ch4.2.1_26P | F: AGTAGGGACGTCATAGTAGG | 1,065,803 | 903 |
|  |  | R: CGAAGTCAATTGTCAGCTTC | 1,066,705 |  |
| 8 | Ch4.2.1_30P | F: GCTATGAATAATGCACGACTG | 1,077,272 | 1,120 |
|  |  | R: GCTTAACCTGTGATCTCCTAG | 1,078,391 |  |
| 9 | Chr4.1.1_2p | F: ATCCGTATACAATGGCGAAAGA | 2,664 | 1,000 |
|  |  | R: CAAGCATTGGTGATTGTTCTCA | 3,663 |  |
| 10 | Chr4.1.1_4p | F: AAACAACAACAACTACGGCTCA | 7,673 | 1,031 |
|  |  | R: ATAAACGGGAACACGAAGAACA | 8,703 |  |
| 11 | Chr4.1.6* | F: TTGTTCTCCCACAAATCACA | 500,078 | 1,065 |
|  |  | R: GAAATTGTCGATGAACATCCGT | 501,142 |  |
| 12 | Chr4.1.6_2p | F: AAAGTTTATGCATGTATGCGGG | 502,693 | 1,042 |
|  |  | R: TAATATGAACCAAAGGGAGCGA | 503,734 |  |

* Markers found to be polymorphic.
