# Supplementary material for: Molecular Mapping of PMR1, a Novel Locus Conferring Resistance to Powdery Mildew in Pepper (Capsicum annuum)
Source: Front Plant Sci. 2017 Dec 8;8:2090. doi: 10.3389/fpls.2017.02090 (PMC5727091; doi:10.3389/fpls.2017.02090)
Supplement: Table S3 — ‘L_Zunla-1' genome-based primer sequences used for polymorphism detection. [file Table3.docx]

**Table S3** ‘L_Zunla-1’ genome-based primer sequences used for polymorphism detection

| **S.No** | **Primer** | **Sequence** | **Position on Chr4** | **Amplicon (bp)** |
| --- | --- | --- | --- | --- |
| 1 | ZL1_1796 | F: CAATTGGGATGGTTCCAACA | 211,796,832 | 1,156 |
|  |  | R: TCAACCTTTACAACCCCCTTT | 211,797,987 |  |
| 2 | ZL1_1826* | F: CGAAGTCATTAAAGTTCATTGGG | 211,826,850 | 1,070 |
|  |  | R: GCAATAAATGCCCTTCCACA | 211,827,919 |  |
| 3 | ZL1_1854 | F: AACCCCCGTGAAATTGAAGT | 211,796,832 | 1,075 |
|  |  | R: CCGAATGCGTTTTAGGTCAA | 211,855,598 |  |
| 4 | ZL1_5685 | F: TGCGTACACAGTGCTCACCA | 215,685,455 | 1,053 |
|  |  | R: CCGTTAAAGCTATACCAGACCAG | 215,686,507 |  |
| 5 | ZL1_5684 | F: CACAAAGATAATGGGGGCAA | 215,684,524 | 914 |
|  |  | R: TATGGTGAGCACTGGGTAAATCT | 215,685,437 |  |
| 6 | ZL1_5678 | F: ATGTTGTTTTGCCCGTTTGA | 215,678,156 | 1,121 |
|  |  | R: TGATCCGGTTGAATGTGCTT | 215,679,276 |  |
| 7 | ZL1_07778 | F: TCTCCGGGCGAAAATAAAGT | 207,778,263 | 1,163 |
|  |  | R: TTGTGCCAGGCTATGCTTTT | 207,779,425 |  |
| 8 | ZL1_07789 | F: GCAATTCGTGATTTTCGAGG | 207,789,731 | 1,185 |
|  |  | R: TGGATTTTCCTAACCGGACA | 207,790,915 |  |
| 9 | ZL1_07799 | F: TTTTCAGGCAAAAGGAAGGC | 207,799,175 | 1,154 |
|  |  | R: CCAATGTGGAAAATGACCCA | 207,800,328 |  |
| 10 | ZL1_10672 | F: TGTCTTTGAAACCGCGAAAC | 210,672,849 | 1,137 |
|  |  | R: TATTTTGTCCCGGTCCCATT | 210,673,985 |  |
| 11 | ZL1_10675 | F: CTTCAATTTCGGCTGCTTCA | 210,675,063 | 1,014 |
|  |  | R: TGGCCAGCTTTCACATGTTT | 210,676,076 |  |
| 12 | ZL1_10691* | F: TCCTGTTTTCTCCCCCTTTT | 210,691,582 | 1,160 |
|  |  | R: CTTTGGCAATATCCCGTTCA | 210,692,741 |  |
| 13 | ZL1_11658* | F: AAAATGCCACATGGCTGAAA | 211,658,167 | 1,056 |
|  |  | R: AGGCCAAAGAAAACCAATGA | 211,659,222 |  |
| 14 | ZL1_11657 | F: AGGCCAAAGAAAACCAATGA | 211,657,279 | 823 |
|  |  | R: TGAAAAGGGGTTGTTGAGAAGA | 211,658,101 |  |
| 15 | ZL1_11079A | F: TTTCCGAAAAACCCGTAGGT | 211,079,184 | 1,156 |
|  |  | R: TTTCCATGTCAAAGGTGGGA | 211,080,339 |  |
| 16 | ZL1_11077 | F: TGGAAACAACCTCTTGCGAA | 211,077,144 | 1,052 |
|  |  | R: TGAATGCACGTTGTTGAGGA | 211,078,195 |  |
| 17 | ZL1_11639 | F: ATTTATCCCAGGCAACGAGA | 211,639,047 | 888 |
|  |  | R: AAATGATGTGCTCCGCTTCT | 211,639,934 |  |
| 18 | ZL1_11628* | F: AGGAACCATTGGGGTCATTT | 211,628,066 | 969 |
|  |  | R: AGGCAATTTTAGCGACCACA | 211,629,034 |  |
| 19 | ZL1_11587 | F: CAGTGGCATATTTGAGCCTTTT | 211,587,312 | 1,002 |
|  |  | R: AATTTTTCGATCTCGACCCC | 211,588,313 |  |
| 20 | ZL1_11532 | F: AAACCGAAATGACTTGTTTCCA | 211,532,831 | 948 |
|  |  | R: AAGGTGGGATAAAGTAGGTTCC | 211,533,778 |  |
| 21 | ZL1_11619 | F: TTAATGGAGACGTGCAAAGG | 211,619,172 | 811 |
|  |  | R: TTTGTGCATGATTCTGTCTTCAG | 211,619,982 |  |
| 22 | ZL1_10768 | F: TCATGTTCTTACATCGCATGG | 210,768,365 | 1,003 |
|  |  | R: AATCTTTATCCTCCGGCCATA | 210,769,367 |  |
| 23 | ZL1_11079B* | F: TTTATCAGGCGTGGAGTTTCA | 211,079,333 | 1,007 |
|  |  | R: TTTCCATGTCAAAGGTGGGA | 211,080,339 |  |
| 24 | ZL1_12987 | F: TTTTGGCAATCGTTTCATCC | 212,987,270 | 1,108 |
|  |  | R: TTGTTCCCCATGAGCATTACA | 212,988,377 |  |
| 25 | ZL1_12384 | F: CCATTAGCAATGGATTGTGGA | 212,384,854 | 902 |
|  |  | R: GAACCTTTCAATTTTCATCGGA | 212,385,755 |  |
| 26 | ZL1_12120 | F: GAAATTACAAACCTGCCTCCAA | 212,120,076 | 1,113 |
|  |  | R: TGGATGCTGAATTTTGTGTCG | 212,121,108 |  |

 * Markers found to be polymorphic.
