# Supplementary material for: Molecular Mapping of PMR1, a Novel Locus Conferring Resistance to Powdery Mildew in Pepper (Capsicum annuum)
Source: Front Plant Sci. 2017 Dec 8;8:2090. doi: 10.3389/fpls.2017.02090 (PMC5727091; doi:10.3389/fpls.2017.02090)
Supplement: Table S4 — Segregation analysis of powdery mildew resistance in ‘PM Singang' population. [file Table4.docx]

**Table S4** Segregation analysis of powdery mildew resistance in the ‘PM Singang’ population

| Population | Number of plants | Phenotype | | Expected ratio | χ^2^ | P-value |
| --- | --- | --- | --- | --- | --- | --- |
|  |  | R | S |  |  |  |
| F_1_ | 21 | 21 | - | - | - | - |
| F_2_ | 80 | 59 | 21 | 3:1 | 0.02 | 0.80 |

R, resistant; S, susceptible.
