# Supplementary material for: Molecular Mapping of PMR1, a Novel Locus Conferring Resistance to Powdery Mildew in Pepper (Capsicum annuum)
Source: Front Plant Sci. 2017 Dec 8;8:2090. doi: 10.3389/fpls.2017.02090 (PMC5727091; doi:10.3389/fpls.2017.02090)
Supplement: Table S5 — Genotyping-by-sequencing of the PMR1 locus. [file Table5.docx]

**Table S5** Genotyping-by-sequencing of the *PMR1* locus

| **SNP marker** | **Position in chromosome 4 (bp)** | ***C. chinense*** | ***C. annuum*** | **‘VK515S’** | ***C. baccatum*** | **‘VK515R’** |
| --- | --- | --- | --- | --- | --- | --- |
| SNP1 | 208,150,769 | G | A | A | G | G |
| SNP2 | 208,150,786 | G | G | G | G | A |
| SNP3 | 208,150,812 | G | G | G | A | A |
| SNP4 | 213,138,206 | A | A | A | G | G |
| SNP5 | 213,138,261 | G | G | G | A | A |
| SNP6 | 213,138,271 | C | C | C | T | T |
| SNP7 | 213,443,584 | G | T | G | T | T |
| SNP8 | 214,126,005 | C | C | C | T | T |
| SNP9 | 214,126,011 | G | G | G | A | A |
| SNP10 | 214,126,045 | G | G | G | A | A |
| SNP11 | 214,126,057 | G | G | G | T | T |
| SNP12 | 214,126,060 | C | C | C | A | A |
| SNP13 | 215,383,249 | T | T | T | A | A |
| SNP14 | 215,383,263 | T | T | C | T | T |
| SNP15 | 215,383,781 | C | C | C | T | T |
| SNP16 | 215,383,782 | A | A | A | G | G |
| SNP17 | 215,383,790 | A | A | A | G | G |
| SNP18 | 215,383,795 | T | T | T | C | C |
| SNP19 | 215,383,827 | T | T | T | C | C |
| SNP20 | 215,383,830 | G | G | G | A | A |
| SNP21 | 215,383,850 | G | G | C | G | G |
| SNP22 | 215,580,751 | G | G | A | G | G |
